# Commentary: Musical hallucinations: review of treatment effects

**DOI:** 10.3389/fpsyg.2015.01885

**Published:** 2015-12-09

**Authors:** Mark D. Griffiths, Angelica B. Ortiz de Gortari

**Affiliations:** International Gaming Research Unit, Psychology Division, Nottingham Trent UniversityNottingham, UK

**Keywords:** musical hallucinations, video games, involuntary musical imagery, Game Transfer Phenomena, involuntary auditory imagery

According to Coebergh et al. (2015), musical hallucinations (MHs) “*are auditory hallucinations characterized by songs, tunes, melodies, harmonics, rhythms, and/or timbres…and that the mechanisms responsible for the mediation of MH are probably diverse”* (p. 2). While some scholars have reported that the prevalence rate of MHs among the general population is at present unknown and/or rare (Vitorovic and Biller, 2013), “involuntary musical imagery” [INMI; i.e., a tune that comes into the mind and repeats without unconscious control (Williamson et al., 2012)] is thought to be more commonplace. For instance, 89% in a Finish sample (*n* = 12,519) reported experiencing it at least once a week (Liikkanen, 2012). Music hallucination prevalence rates among various groups have been reported including obsessive-compulsive disorder patients (41%; Hermesh, 2004), elderly people with auditory problems (2.5%; Cole et al., 2002), and general hospital setting patients (0.16%; Fukunishi et al., 1998). Possible etiological factors include otological factors (e.g., hearing loss), intoxication, brain injury, epilepsy, and psychiatric disorders (Cope and Baguley, 2009; Coebergh et al., 2015).

Although Coebergh and colleagues described MHs, they were not explicitly defined. In other reviews, Woo et al. (2014) defined MHs as “*complex auditory perceptions in the absence of an external acoustic stimulus and are often consistent with previous listening experience”* (p. 1) whereas Vitorovic and Biller (2013) noted that MHs “*represent a specific form of auditory hallucinations whereby patients experience formed songs, instrumental music, or tunes, without an external musical stimulus”* (p. 1). Williams (2015) provided a classification of INMI and noted they cover a number of different types of involuntary musical experience (including MHs). Despite the lack of detailed definition, it is known that MHs occur within the context of an individual's culture and are often viewed by those experiencing them as intrusive and sometimes unpleasant (Cope and Baguley, 2009; Vitorovic and Biller, 2013).

As far as the present authors are aware, no review paper examining musical hallucinations has ever included papers referring to musical hallucinations arising from playing video games. The earliest report in the psychological literature is by Spence (1993) who reported the case of a 20-year-old female patient with a family history of psychosis. She presented with persecutory delusions, suicidal ideation, violent behavior, and third-person auditory hallucinations comprising 48 h of constant MHs from the *Mario Bros* videogame that developed into delusional thoughts. No drugs were found in her urinary system and her EEG was normal when MHs occurred. The MHs from the videogame decreased within 48 h of treatment (via antidepressants/neuroleptics).

More recently, a series of papers by the present authors examined Game Transfer Phenomena (GTP). GTP research has demonstrated how the videogame can keep on playing even after the game has been turned off. GTP are non-volitional phenomena (e.g., altered perceptions, automatic mental processes, and involuntary behaviors) (Ortiz de Gortari and Griffiths, 2015). Analysis of over 1,600 gamers' self-reports have shown that videogame playing can lead to (i) perceptual distortions of physical objects, environments, and/or sounds, (ii) misperceptions of objects and sounds that are similar to those in the videogame, (iii) interpretation of events in real life contexts that utilize the logic of the videogame, (iv) ghost perceptions and sensations of images, sounds, and tactile experiences, and (v) involuntary actions and behaviors based on experiences from the videogame (Ortiz de Gortari et al., 2011, 2015; Ortiz de Gortari and Griffiths, 2014a,b,c, 2015).

One study (Ortiz de Gortari and Griffiths, 2014b) specifically examined auditory GTP experiences. Gamers' experiences identified as GTP in one or more modalities (e.g., visual, auditory) were collected from 60 online videogame forums over 7 months. Of these, there were 192 auditory experiences from 155 gamers collected. The largest numbers of experiences (90%) were identified as involuntary auditory imagery. This manifested as hearing music (*n* = 73), sound (*n* = 83), or voices from within the game (*n* = 12). Some experiences were triggered by external cues associated with the videogame, while others were not. Experiences with music included hearing high pitch music in addition to calm and classical music.

Music from the videogames was usually experienced persistently, while sound effects or voices appeared to occur more episodically. Hearing the music persistently provoked sleep deprivation, annoyance, and uncertainty. When the music was re-experienced very vividly, the gamers attributed them to external sources associated with the videogame. More specifically, when auditory cues were associated with adverse videogame content, they resulted in such things as hearing sounds coming from objects around them or hearing their own inner speech in a videogame character's voice (Ortiz de Gortari and Griffiths, 2014a). In many cases, the gamers said that they had been playing intensively (i.e., either playing long sessions or playing frequently). Previous studies have linked hearing music in absence of auditory stimuli with the recent or repeated exposure to music (e.g., Gardner, 1985; Gerra et al., 1998; Hyman et al., 2012). One gamer said that he heard the sound of music coming out from the speakers so he stood up to check them, while another heard music from *Pokémon* when vacuuming. It also appears that musical hallucinations can cross sensory modalities. For instance, some gamers have reported hearing music while seeing images from the videogame (Ortiz de Gortari and Griffiths, 2014a). An online survey about GTP with a convenience sample of 2,362 gamers found that hearing music from videogames when not playing were the more prevalent (74%) than hearing sounds (65.0%) or voices (46%) when not playing (Ortiz de Gortari and Griffiths, Unpublished manuscript).

Based on what is known empirically, we would conclude that (i) MHs from videogame playing—although not well documented—appear to be relatively commonplace among gamers, and prevalence appears to be higher than found in other populations, (ii) individual interpretation of MHs from videogames are influenced by the meanings and uses of auditory cues in the videogames, (iii) MHs can manifest beyond one sensory modality and have been reported across-sensory channels (e.g., hearing music while seeing ghost images from the game), (iv) those researching in the field of MHs and INMI appear to have overlooked the literature on these phenomena related to videogame playing, (v) better definitions and further research are needed for MHs, and a distinction between MHs and INMI is required, as etiological issues have not been systematically studied among video game players.

## Author contributions

MG thought of the idea for the paper and wrote the first draft of the paper. AO then added material to the draft. Both authors then edited the paper down.

### Conflict of interest statement

The authors declare that the research was conducted in the absence of any commercial or financial relationships that could be construed as a potential conflict of interest.
